# Moral harassment in healthcare through the lens of Diego Gracia’s deliberative bioethics: A practical framework for institutional reform

**DOI:** 10.1016/j.clinsp.2026.101107

**Published:** 2026-09-07

**Authors:** Fábio Roberto Cabar

**Affiliations:** Division of Obstetrics, Hospital das Clínicas da Faculdade de Medicina da Universidade de São Paulo, São Paulo, SP, Brazil

## Introduction

Moral harassment, frequently conceptualized in organizational psychology as workplace mobbing or systemic psychological bullying, is defined as a deliberate, repetitive, and prolonged pattern of hostile, intimidating, or humiliating behaviors.^1^ Rather than isolated episodes of interpersonal friction, occasional administrative disagreements, or a single instance of poor communication, moral harassment manifests as a sustained campaign of psychological degradation. These actions intentionally target an individual’s professional competence, social standing, or emotional resilience, progressively eroding self-esteem and acting as a potent catalyst for severe psychiatric morbidity, including major depressive disorder, generalized anxiety, and burnout syndrome.^1^

While psychological violence occurs across all employment sectors, the healthcare environment exhibits a unique structural susceptibility to these toxic dynamics. Recent peer-reviewed studies highlight that competitive medical cultures and extreme operational workloads create highly fertile ground for institutional abuse.^2^ For instance, recent investigations into the mental health of medical trainees in Brazil reveal that a vast majority of residents report frequent exposure to public humiliation, systemic isolation, or arbitrary punitive measures by superiors.^2^

• This pronounced vulnerability within healthcare settings stems from three deeply entrenched structural and cultural characteristics:
• Rigid, Vertical Power Hierarchies: Power structures within clinical medicine and hospital administration are strictly top-down. Trainees (students, interns, and residents) and junior staff depend entirely on supervising attending physicians, department heads, and directors for clinical training, career advancements, fellowships, and essential professional references.
• The Normalization of Stress as a “Rite of Passage”: A pervasive historical subculture frequently conflates chronic sleep deprivation, extreme emotional strain, and harsh interpersonal friction with necessary professional socialization. Senior practitioners often minimize hostile behaviors as traditional, time-tested methods to “toughen up” young clinicians.
• High-Stakes, High-Friction Environments: Chronic understaffing, heavy patient volumes, and the continuous psychological weight of managing life-and-death clinical scenarios create a permanent state of high stress. In the absence of healthy institutional outlets, systemic burnout frequently manifests as vertical or lateral interpersonal aggression.

### Diego Gracia’s framework: from hierarchy to deliberative practice

To analyze this phenomenon structurally, the framework of Spanish bioethicist Diego Gracia provides an invaluable philosophical and practical tool. Gracia proposes a two-level hierarchy of bioethical principles.^3^ Level 1, known as the *Ethics of Minimums*, comprises the principles of Non-maleficence and Justice. These principles represent universal, coercive, and legally enforceable duties that govern basic human coexistence. They dictate what we owe to others regardless of personal choices or specific philosophical alignments: the absolute duty not to cause harm and the duty to treat every person as an equal moral agent.^3^ Conversely, Level 2, or the *Ethics of Maximums*, encompasses the principles of Autonomy and Beneficence. These are intrinsically linked to individual life projects, personal value systems, and the subjective pursuit of professional fulfillment or happiness.^3^^,^^4^

A common oversimplification reduces Gracia’s framework to a mechanical binary rule, assuming that a lower-level preference can never conflict with a higher-level one without an automatic resolution. However, Gracia’s central contribution to modern applied ethics is his *deliberative method*, a structured process for weighing conflicting values in specific, complex cases where absolute rules fail to capture the full human reality.^4^ Moral deliberation requires a prudent, reasonable, and systematic analysis that treats all involved parties as valid moral agents. Gracia outlines this mental workflow across three core operational dimensions or vectors:

1. The Cognitive Vector (Facts): Establishes objective, verified clinical, institutional, and organizational data, stripping the scenario of defensive biases or emotional distortion.
2. The Affective Vector (Values): Maps out the conflicting values held by the different moral agents involved, identifying which fundamental principles are being threatened or compromised.
3. The Operative Vector (Duties): Identifies the full spectrum of possible courses of action, systematically evaluating and rejecting extreme or imprudent options to determine the optimal intermediate course that honors fundamental moral requirements.^4^

Through systematic deliberation, an institution can effectively distinguish legitimate pedagogical or professional rigor from abusive behavior. Demanding feedback, objective critiques, and rigorous evaluations are essential to building clinical competence and ensuring patient safety. True professional rigor is objective, transparent, respectful, and focused entirely on the trainee's development. It honors Level 1 duties by protecting patients from harm while constructively guiding the student.

Conversely, moral harassment leverages structural hierarchy to intentionally mock, isolate, or degrade a vulnerable professional. While high educational standards represent a pursuit of professional excellence (Beneficence – Level 2), deliberation demonstrates that they cross the line into absolute ethical failure the moment they rely on humiliation, which directly violates the non-negotiable duty of Non-maleficence (Level 1).

## Extended case illustration: the method of moral deliberation in action

To demonstrate how Diego Gracia’s deliberative vectors operate to resolve complex institutional conflicts, consider the following anonymized, well-developed scenario within a teaching hospital:

The Scenario: A first-year internal medicine Resident (R1) makes a minor administrative error on a patient admission chart, which does not result in clinical harm. In response, the supervising attending physician routinely singles out the R1 during public grand rounds over the next three weeks, using derogatory language and labeling the resident “clueless” and “a liability to the department” in front of peers, nurses, and patients. Furthermore, as a self-described “educational punishment” to enforce discipline, the attending forces the resident to log consecutive, non-pedagogical 36-hour shifts dedicated exclusively to repetitive bureaucratic paperwork. The resident begins showing signs of severe anxiety, acute insomnia, and eventually commits a secondary clinical error during a medication prescription due to profound sleep deprivation.

Applying Gracia’s deliberative framework step-by-step reveals the following path:

• Fact Assessment (Cognitive Vector): The deliberation gathers objective data. The administrative error caused no patient harm. The attending’s response was repetitive, public, verbal, and psychological, combined with physical exhaustion tactics (36-hour shifts). The resident’s secondary clinical error was directly linked to documented sleep deprivation. These verified facts demonstrate that the behavior is not an isolated instance of critical feedback, but a systematic pattern of psychological degradation.
• Value Mapping (Affective Vector): The values in conflict are identified. The attending physician claims their actions are driven by “rigorous clinical education”, “discipline”, and “ensuring department efficiency” (a misguided pursuit of Level 2 Beneficence). The resident appeals to “personal dignity”, “psychological safety”, and “the right to a safe learning environment” (Level 1 Non-maleficence and Justice). Deliberation reveals that the attending's method actively compromises both the resident's health and patient safety. Therefore, the purported educational benefit is invalidated because causing systematic psychological harm (Maleficence) and abusing structural hierarchy to deny equal human dignity (Injustice) directly violates universal Level 1 minimums.
• Examining Courses of Action (Operative Vector): The deliberators outline the spectrum of choices to find a prudent, intermediate outcome:
• Extreme Course A (Institutional Passivity): Forcing the resident to endure the behavior to maintain departmental hierarchy or avoid conflict with a senior attending. This completely fails the absolute duty of non-maleficence and perpetuates systemic injustice.
• Extreme Course B (Immediate Summary Termination): Terminating the attending physician instantly without due process or formal investigation, which could trigger operational chaos, structural injustice, or legal countersuits.
• The Intermediate/Optimal Prudent Course: Immediately reassigning the resident to a different clinical team to safeguard their physical and mental well-being (fulfilling the non-negotiable duty of non-maleficence). Concurrently, an independent institutional compliance committee conducts a formal review of the attending's conduct, enforces mandatory leadership training, and applies proportionate administrative and structural penalties (fulfilling Justice). This intermediate action removes the harm immediately while preserving procedural justice for all parties.

## Local context: the Brazilian socio-legal framework

In the specific context of Brazilian healthcare and medical education, these ethical boundaries are heavily reinforced by definitive legal frameworks. Brazilian Federal Law (*Lei* nº 13.185/2015) explicitly codifies the concept of systematic intimidation (*intimidação sistemática*, or bullying), mandating that educational and labor institutions implement proactive, continuous measures to prevent, diagnose, and curb psychological violence.^5^ Furthermore, regional regulatory bodies, such as the Regional Medical Council of the State of São Paulo (CREMESP), have published specific guidance manuals to address moral harassment among young physicians and trainees, establishing clear ethical boundaries for residency supervisors.^6^

For major academic medical centers, such as the Hospital das Clínicas of the University of São Paulo Medical School (HCFMUSP), operating within a highly specialized, high-pressure tertiary environment can exacerbate these systemic pressures. While large-scale, multi-center contemporary data regarding individual Brazilian institutions remain scarce, this limitation itself underscores an urgent need for multi-center local research. Acknowledging these structural challenges within the Brazilian legal context allows institutions to move past outdated cultural acceptances and implement modern compliance standards aligned with federal anti-bullying legislation.

## Interprofessional dynamics and epistemic injustice

Beyond medical residency, moral harassment and interpersonal disrespect are heavily anchored in the abuse of power across diverse healthcare professions. For instance, a seminal study involving nursing professionals in Brazil revealed that 47% of respondents had witnessed instances of profound disrespect from superiors in their workplace.^7^ It is vital to clarify that witnessing disrespect, while distinct from being the direct target of personal, repeated harassment, is deeply damaging to the institutional fabric. A toxic environment where disrespect is tolerated compromises the collective psychological safety of the entire healthcare team. In these scenarios, the violation of the principle of Justice is clear: the institutional system fails to protect its professionals, allowing formal organizational hierarchies to supersede fundamental human dignity.

A parallel degradation of ethical principles can occur within the physician-patient relationship, where historical authoritarianism can nullify patient autonomy. However, a key distinction must be made regarding harassment: a single instance of poor communication, paternalistic language, or cultural insensitivity, while problematic, does not meet the legal or ethical criteria for systematic moral harassment.

Letting structural communication barriers become permanent can be precisely understood through the framework of *epistemic injustice*, as developed by Miranda Fricker.^8^ Epistemic injustice occurs when a speaker is wrongfully deflated in their credibility due to identity prejudices or systemic power differentials.^8^ When healthcare professionals systematically employ inaccessible technical jargon, dismiss patient accounts of their own symptoms, or intentionally withhold vital clinical information, they maintain an abusive control over the clinical course. This behavior treats patients as if they are incapable of understanding or contributing to their own health choices. This disregard for the patient's human condition, frequently exacerbated by deep socioeconomic disparities in the Brazilian public healthcare system, represents a failure of Non-maleficence that invalidates the patient's capacity as a knower.

## Actionable institutional recommendations

Eradicating moral harassment requires moving beyond general calls for “codes of conduct” and transitioning from a punitive culture to a proactive culture of learning. Healthcare organizations must establish specific, measurable, and structurally independent policies:

1) Mandatory Annual Deliberative Ethics Rounds: Every clinical department and residency program should implement mandatory, case-based ethics sessions utilizing Diego Gracia's deliberative method. These rounds must analyze anonymized scenarios of hierarchical and interprofessional conflict, training staff to distinguish between constructive clinical feedback and abusive behavior. Success should be measured by documented departmental attendance and pre-and-post-intervention psychological safety surveys.
2) Independent Ombudsperson and Anti-Retaliation Safeguards: Institutions must establish a secure, anonymous reporting infrastructure managed by an independent ombudsperson operating completely outside departmental or hospital hierarchies. This system must include strict, legally binding protections to ensure zero retaliation against whistleblowers, trainees, or staff members who report misconduct.
3) Formal Ethical Deliberation and Anonymous Publishing: Every verified report of moral harassment should undergo a structured ethical review by an independent compliance committee. To foster long-term institutional transparency and collective learning, the final resolutions, intermediate courses of action, and corrective measures applied should be published anonymously across the organization on a semi-annual basis.

## Conclusion

Moral harassment is not merely unkind; it violates the most fundamental duties that any healthcare professional owes to another person – the duty not to harm and the duty to treat each person as an equal. Eradicating this systemic issue requires an unwavering recognition that the Ethics of Minimums is entirely non-negotiable. The true ethical commitment of 21st century medicine is not solely technical or clinical; it is the absolute duty to ensure that, in our collective pursuit of healing or teaching, Non-maleficence and Justice remain the unbreakable, practical pillars of daily clinical life.

## CRediT authorship contribution statement

**Fábio Roberto Cabar:** Conceptualization, Writing – original draft.

## Declaration of competing interest

The authors declare no conflicts of interest.

## Data Availability

Not applicable.
